# A CT scan protocol for the detection of radiographic loosening of the glenoid component after total shoulder arthroplasty

**DOI:** 10.3109/17453674.2013.869653

**Published:** 2014-02-25

**Authors:** Thomas Gregory, Ulrich Hansen, Monica Khanna, Celine Mutchler, Saik Urien, Andrew A Amis, Bernard Augereau, Roger Emery

**Affiliations:** ^1^Department of Orthopaedic Surgery, University Paris Descartes, European Hospital Georges Pompidou,APHP, Paris,France; ^2^Mechanical Engineering, Imperial College London, London; ^3^Department of Radiology, St. Mary’s Hospital, London, UK; ^4^Department of Radiology, University Paris Descartes, European Hospital Georges Pompidou, APHP, Paris; ^5^Unité de Recherché Clinique Paris Centre, University Paris Descartes, European Hospital Georges Pompidou, APHP, Paris, France; ^6^Division of Surgery and Cancer, Imperial College London School of Medicine, London; ^7^Department of Orthopaedic Surgery, St. Mary’s Hospital, London, UK.

## Abstract

**Background and purpose:**

It is difficult to evaluate glenoid component periprosthetic radiolucencies in total shoulder arthroplasties (TSAs) using plain radiographs. This study was performed to evaluate whether computed tomography (CT) using a specific patient position in the CT scanner provides a better method for assessing radiolucencies in TSA.

**Methods:**

Following TSA, 11 patients were CT scanned in a lateral decubitus position with maximum forward flexion, which aligns the glenoid orientation with the axis of the CT scanner. Follow-up CT scanning is part of our routine patient care. Glenoid component periprosthetic lucency was assessed according to the Molé score and it was compared to routine plain radiographs by 5 observers.

**Results:**

The protocol almost completely eliminated metal artifacts in the CT images and allowed accurate assessment of periprosthetic lucency of the glenoid fixation. Positioning of the patient within the CT scanner as described was possible for all 11 patients. A radiolucent line was identified in 54 of the 55 observed CT scans and osteolysis was identified in 25 observations. The average radiolucent line Molé score was 3.4 (SD 2.7) points with plain radiographs and 9.5 (SD 0.8) points with CT scans

(p = 0.001). The mean intra-observer variance was lower in the CT scan group than in the plain radiograph group (p = 0.001).

**Interpretation:**

The CT scan protocol we used is of clinical value in routine assessment of glenoid periprosthetic lucency after TSA. The technique improves the ability to detect and monitor radiolucent lines and, therefore, possibly implant loosening also.

Glenoid loosening is the main complication of total shoulder arthroplasty (TSA). In a recent analysis that included 33 clinical studies and 2,540 shoulder arthroplasties from 1996 to 2005, the rate of aseptic loosening was reported to be 39%; of these, 83% involved the glenoid component (Bohsali et al. 2006).

In current practice, patient evaluation after TSA is based on successive clinical and radiographic assessments with plain and profile radiographs. Radiolucent lines are often seen around the glenoid implant on plain radiographs, and they are thought to be linked to glenoid loosening. The mean rate of radiolucent lines has been reported to be 80% (Bohsali et al. 2006) in a series with more than 10 years of follow-up, which indicates the scale of the loosening problem. The reported occurrence of radiolucent lines also varies greatly between published studies (from 0% to 100%) (Cofield 1984, Barrett et al. 1987, Torchia et al. 1997, Boileau 2000, Mileti et al. 2004, Desmukh et al. 2005, Martin et al. 2005, Szabo et al. 2005). Thus, it appears that the radiolucent lines seen on plain radiographs have only a tenuous link with loosening. One possible reason for this is that radiolucent lines are not always seen, even when the implant really is loose.

Yian et al. (2005) used computed tomography (CT) to identify and assess radiolucencies in the glenoid fixation for the purpose of evaluating component loosening. However, they concluded that major artifacts (caused by the metallic humeral head) severely blurred the images, preventing reliable analysis of the implant fixation.

We describe a simple and reproducible patient-positioning protocol that moves the metallic humeral head out of the CT scan acquisition plane, reducing metal artifacts and thereby providing clear images for analysis and monitoring of radiolucent lines adjacent to the glenoid fixation.

We quantitatively assessed the usefulness of the protocol for evaluation of glenoid component periprosthetic radiolucency and compared it to that of standard methodology using plain-film radiographs to assess loosening. A secondary aim was to assess radiolucent lines in 11 patients to demonstrate the feasibility and the usefulness of the method.

## Patients and methods

**Figure 1.**
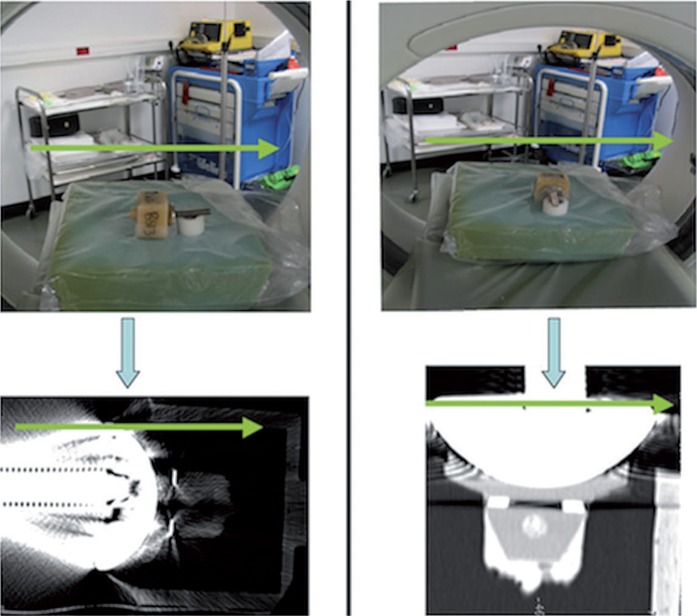
Shoulder prosthesis mounted in 2 different positions in a CT scanner. Humeral component placed against an all-polyethylene glenoid component and the prosthesis aligned perpendicularly (left part of figure) and axially (right part of figure) relative to the axis of the scanner. The opening of the CT scanner is shown, and indicates the orientation of the specimen relative to the scanner. The glenoid component was fixed into a box using PMMA bone cement. The bottom part of the figure shows CT scan images of the artificial total shoulder replacement oriented perpendicular to, and parallel to, the 2D CT acquisition plane. The green arrows in the figure indicate the orientation of the scanner, by which the alignment of the implant with respect to the CT acquisition plane can be evaluated.

To demonstrate the principle of the proposed clinical imaging method, first an ex vivo shoulder prosthesis was CT scanned while mounted in 2 different orientations in a scanner (Figure 1). The implant included a cobalt-chromium alloy humeral component (Aequalis Shoulder; Tornier Inc.). This was articulated against an all-polyethylene glenoid component with a fixation keel. The glenoid component was fixed into a box using PMMA bone cement (Simplex Rapid; Kemdent). The prosthesis was placed on the central axis of a CT scanner, oriented either transversely to the scanner axis or along the scanner axis. When the prosthesis was arranged axially, the CT acquisition plane (the CT slice) was parallel to the glenohumeral joint space. When the prosthesis was oriented transversely to the scanner axis, the CT acquisition plane was perpendicular to the glenohumeral joint space and cut through both the humeral and glenoid components. This latter orientation corresponds to standard clinical examination CT protocols.

**Figure 2.**
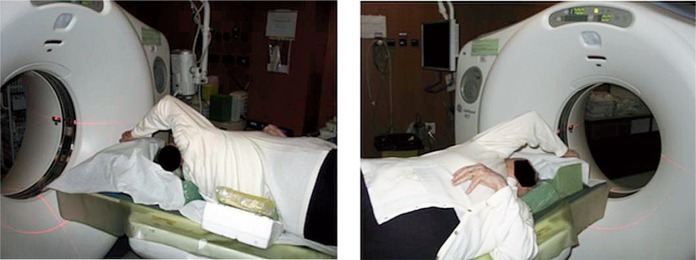
Position of the patient in the CT scanner as used in this study: patient in lateral decubitus or in three-quarter decubitus, allowing the scapula to tilt, with shoulder in maximal flexion.

In the standard patient position in the CT scanner, the patient lies supine with the arms at the side of the body, resulting in the glenohumeral joint being transverse to the CT acquisition plane. As noted in a previous study (Yian et al. 2005), the resulting image quality is severely reduced due to the artifacts mentioned. In the clinical setting, this issue can be resolved by taking advantage of the mobility of the shoulder. During shoulder abduction and flexion, the tilt of the scapula and the spinal side flexion allow the glenoid to be positioned in the transverse body plane (Crosbie et al. 2008, Fayad et al. 2008). This positioning results in a near-axial orientation of the glenohumeral joint in the CT scanner. Figure 2 illustrates the patient position adopted in this study to reduce the artifacts: patient in lateral decubitus to three-quarter decubitus position, allowing the scapula to tilt and the shoulder to be in maximal forward flexion.

**Figure 3.**
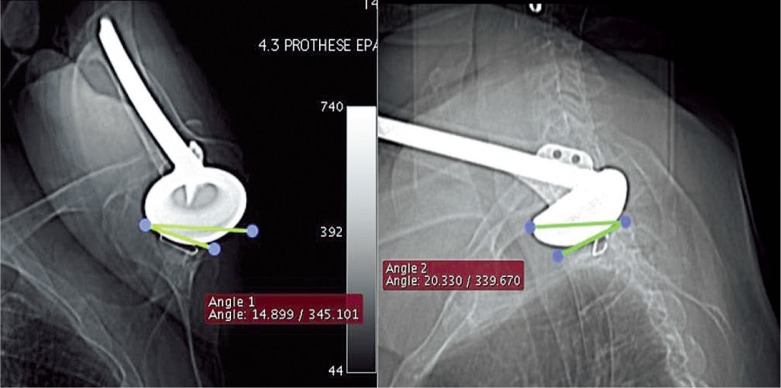
Coronal and sagittal scout views of the shoulder with the most severely limited active flexion angle of the series and also indicating the angle (non-ideal alignment) between the glenoid orientation and the CT-scan acquisition plane.

Scout views of the shoulder in 2 different planes (Figure 3) were inspected to determine how closely the glenoid orientation matched the CT acquisition plane, and to re-adjust the patient position if needed. Patient position was maximized to ensure that the orientation of the glenoid component was near-parallel to the CT scanner axis. Any position in which the humeral head was placed above the glenoid component was considered appropriate. To minimize radiation exposure, the acquisition field of the CT scanner was limited to the glenoid region of the scapula. The resulting radiation dose was assessed by the Radiation and Protection Advisor at APHP, using CT-Expo V 1.6 17 (Stamm and Nagel 2002) software, as being 2.6 mSv—which is equivalent to 1 year of natural background radiation. In comparison, radiation doses from plain radiographs and conventional shoulder CT scans were assessed as being 0.08 mSv and 3–3.5 mSv, respectively.

**Figure 4.**
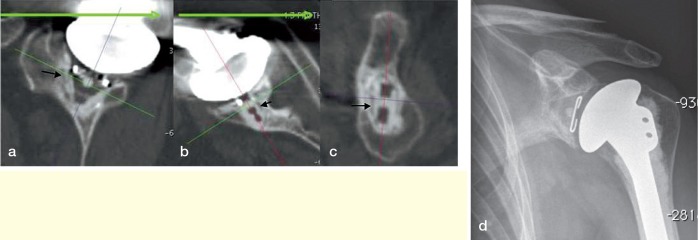
Radiolucent line at the cement-bone interface (black arrows) visualized by using the described protocol, in the sagittal plane of the glenoid implant (a), in the axial plane of the middle part of the implant (b), in the coronal plane of the implant, passing through the keel hole (c). Plain radiograph of the same shoulder for comparison (d).

A patient CT scanner (Table 1) was used to obtain 1-mm-thick contiguous slices with in-plane resolution of 11.02 lp/cm × 10.69 lp/cm (0.45 × 0.47 mm), calculated using modular transfer function (MTF). Reconstruction in 3 orthogonal planes (Figure 4) was used to facilitate glenoid visualization and periprosthetic assessment.

**Table 1.** CT settings (64-bit data acquisition system; Light Speed VCT; GE)

Scan options	Helical mode
U (kV)	140
I (mA)	55
t (s)	1
N*hcol (mm)	20
TF (mm)	10.6
hrec (mm)	1.3
P	0.5

The study involved 11 patients (mean age 70 (48–83) years, 10 women) who had undergone glenohumeral joint replacement surgery for osteoarthritis between June 2002 and January 2005. The mean follow-up was 45 (33–63) months. 3 patients had unsatisfactory results (pain and reduced range of motion) at the last outcome assessment. Standard-sized Smith and Nephew Neer II Anatomic shoulder components were implanted using a superior approach and a first-generation cementing technique. 2 sizes of humeral heads were used: 15 mm in 7 patients and 22 mm in 4 patients.

**Figure 5.**
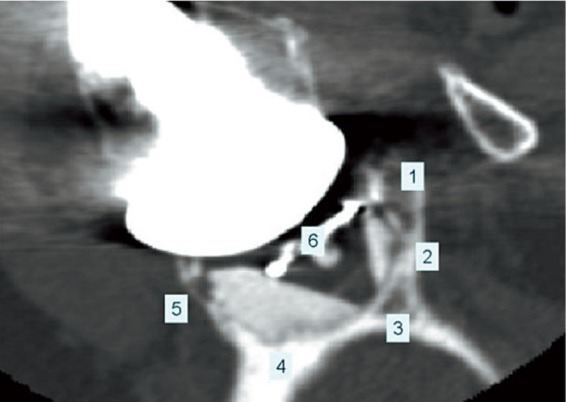
Coronal view of the glenoid bone including the implant. The numbers indicate the 6 zones used in the Molé Score to assess the level of radiolucent lines in the fixation of the glenoid component. Each zone is scored between 0 and 3 points according to the level of radiolucent lines observed and the Molé Score is the sum of these scores. Zone 1: fixation area of the superior part of the glenoid component base plate; Zone 2: fixation area of the superior part of the keel; Zone 3: fixation area of the tip of the keel; Zone 4: fixation area of the inferior part of the keel; Zone 5: fixation area of the inferior part of the glenoid component base plate; Zone 6: fixation area of the central part of the glenoid component base plate.

Plain radiographs (AP view and axillary view) were also taken at the last patient evaluation. These radiographs, rather than conventional shoulder CT scans, formed the control group. The reason for this choice was that plain radiographs are still the standard when assessing loosening, and Yian et al. (2005) have shown that conventional CT scans suffer from severe artifacts. Thus, we could not justify exposing the patients to another CT radiation dose in addition to the dose they would receive from our technique. The imaging studies (radiographs and CT scans) were anonymized, randomized, and assessed by 5 different observers (2 orthopedic surgeons and 3 musculoskeletal radiologists) for radiolucent line analysis. For each radiological examination, the glenoid fixation was divided into 6 areas in the sagittal plane (Figure 5) and an evaluation of radiolucent lines in each area was made according to the Molé criterion (Molé et al. 1999). CT scans were also assessed for osteolysis, which we defined as a radiolucent line of more than 3 mm in both thickness and length, and located at the bone-cement interface.

### Statistics

The radiolucent line Molé scores were analyzed in relation to the imaging technique used (radiographs or CT scan) and the observers, using a repeated-measures analysis of variance (ANOVA). Significance was set at p < 0.05. In order to define difference in data dispersion between CT scan and radiography, the mean intra-subject score variability was estimated using ANOVA for each imaging technique. Statistical analysis was performed using the R software package (R Development Foundation for Statistical Computing, Vienna, Austria).

### Ethics

Follow-up CT scanning as described in Patients and methods is part of our routine patient care at APHP, and the patient position protocol was approved by the local Radiation Protection Advisor and subsequently by the Institutional Review Board.

## Results

When the face of the glenohumeral joint was oriented perpendicular to the acquisition plane of the CT scan, major artifacts, caused by the presence of the metallic humeral head, obscured the image around the glenoid implant fixation. In contrast, when the glenohumeral joint was oriented parallel to the CT acquisition plane, these artifacts were dramatically reduced if not eliminated (Figure 1).

The Constant-Murley shoulder outcome score used in postoperative assessment—which includes shoulder pain, shoulder function, range of motion, and strength measures—varied from 12 to 88 at the latest follow-up. 3 of the patients had stiff joints with less than 50° of active flexion; however, no association between this stiffness and any underlying reason could be found. The average active flexion angle of all patients was 108° (40–160). No scapulo-thoracic or thoracic spine diseases were detected before CT scanning.

Evaluated from the 2 scout views, the mean angle between the glenoid plane and the CT acquisition plane was 23° (SD 8) for the 11 patients. Positioning of the patient in the CT scanner in the proposed position was feasible for all 11 patients, including the 3 patients with stiff glenohumeral joints (Figure 3). Alignment of the glenoid and the acquisition planes resulted in artifacts in only small regions of the superior and posterior parts of the glenoid fixation, while most of the fixation was clearly visible (Figure 4).

Figures 4 and 5 show the ability of the CT methodology to visualize radiolucent lines around the glenoid implant fixation. Radiolucent lines in 11 patients were scored according to the Molé scoring system by 5 observers (with a total of 55 observations). Most notably, a radiolucent line was present in 54 of the 55 observations, and in 26 of the cases this line was wider than 1 mm. The average Molé score was higher in the CT scan group than in the radiography group (p = 0.001), and both the SD and the mean intra-observer variance were lower in the CT scan group than in the radiography group (p = 0.001) (Table 2).

**Table 2.** Comparison of plain radiographs and CT scan results from 55 observations in total (11 patients each observed by 5 observers)

	Radiolucent lines ^**a**^		Osteolysis
	Mean (SD) [range]	Mean intra- observer variance	No. of observations
Plain radiographs	3.4 (2.7) [0–18]	57	6
CT scan	9.5 (0.8) [1–18]	18	25

**^a ^**Molé score: points

**Figure 6.**
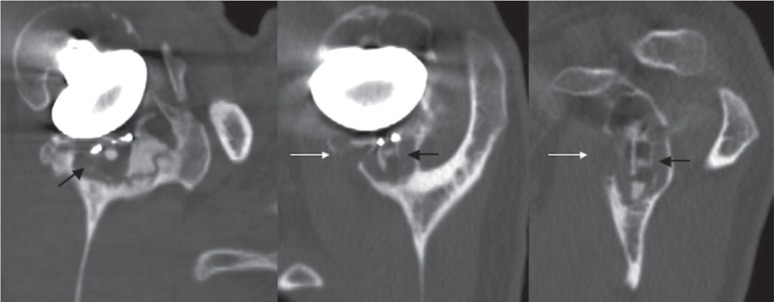
Cancellous bone osteolysis (indicated by the black arrows) and cortical bone osteolysis (indicated by the white arrows) visualized using the described protocol, in the sagittal plane of the glenoid implant (left), in the axial plane of the middle part of the implant (middle), in the coronal plane of the implant, passing through the keel hole (right).

The radiolucent lines detected were always located at the bone-cement interface. In 25 of the 55 observations, osteolysis was present around the keel of the implant (Figure 6).

## Discussion

Our CT protocol allowed the glenoid fixation to be visualized in 3D with few artifacts, and thus enabled a more accurate analysis of radiographic loosening. Compared to plain radiographs, radiographic loosening scores were 3 times higher using this protocol, while intra-observer variability and standard deviation were much lower. Osteolysis was identified in 25 of the 55 observations, as compared to just 6 of 55 when using plain radiographs.

The improved assessment of radiographic glenoid loosening from the CT was achieved by aligning the CT and glenoid planes. Even with stiff postoperative glenohumeral joints, the protocol described, using scapula tilt and spine side flexion, allowed good visualization of the glenoid fixation. Evaluation of patients before CT scanning is necessary to ensure that patients are able to adopt an adequate position, particularly in those with diseases of the scapulothoracic space or of the spine.

In an in vitro study, Gregory et al. (2009) compared glenoid implant fixation (using the CT scan protocol outlined in our study) to observations using microscopy of physically sectioned specimens, and showed that the radiolucencies on CT scans did correspond to implant debonding.

The incidence of radiolucencies as evaluated from plain radiographs varies between 0% and 100% (Cofield 1984, Barrett et al. 1987, Torchia et al. 1997, Mileti et al. 2004, Desmukh et al. 2005, Martin et al. 2005, Szabo et al. 2005, Yian et al. 2005). This large variation reflects the difficulty in using standard radiography for the purposes of evaluating implant loosening. Our small series included only 3 painful shoulders. Of these, 2 performed well immediately after surgery, with low Molé scores, but at the time of the latest follow-up they had become painful—with high Molé scores of 18 and 14. Interestingly, only in these 2 (of 11 cases) had the cortices been breached (Figure 6). The third shoulder was painful immediately after the index surgery; this was not reflected in the radiographic assessment, which showed a Molé score of just 7 and no cortical involvement. Perhaps these results indicate that pain is associated with progressive radiolucencies only when these extend to include the cortices, while shoulders that are painful immediately following the surgery have a different etiology. Although the CT protocol offers an improved ability to characterize radiolucencies, the patient series was too small to make any conclusions regarding the association between radiographic loosening and clinical outcomes such as pain or clinically loose implants. Future studies are needed to establish symptomatic radiolucencies by relating radiolucencies to loose implants. However, the main aim of the present study was not to determine these relationships but to provide a tool that would enable future studies to undertake such investigations.

Previous studies have found radiolucent lines at the implant-cement interface (Nyffeler et al. 2003) and others at the cement-bone interface (Torchia et al. 1997). The latter finding is consistent with our findings. The radiodensity of the polyethylene implant was relatively low and, as seen from Figures 4 and 5, the implant appeared dark on the CT images. Therefore, even if a radiolucent line was present at the implant-cement interface, it would be difficult to differentiate such dark lines from the implant. The method must be developed further to detect any radiolucencies that may be present at the implant-cement interface.

Based on histological analysis of 3 retrieved glenoid components, Wirth et al. (1999) pointed out the role of osteolysis caused by polyethylene particles in aseptic loosening of the glenoid component. Wear particle granuloma has also been proposed as a cause of glenoid loosening, through indirect data from another study of retrieved components (Scarlat and Matsen 2001) and a Finite Element study (Hopkins et al. 2007). We detected osteolysis in 25 of the 55 observations, supporting the hypothesis that biological phenomena play a role in glenoid loosening. Our CT scan protocol provides an advantageous method for detection and monitoring of osteolysis in patients.

There are indications that new CT reconstruction software would also reduce metal artifacts and this may be an alternative technique to the one suggested in our study. However, we have been unable to find any studies that have evaluated the efficacy of these new techniques in the context of shoulder arthroplasty. The only 2 papers that did use CT scanning (Yian et al. 2005 and Arnold et al. 2011) both described substantial difficulties due to metal artifacts.

The CT protocol suggested here makes identification of radiolucencies in advance of them being detectable on plain radiographs more likely, and its use should be considered in total shoulder arthroplasty follow-up studies.
